# Eastern Provincial Medical and Surgical Association

**Published:** 1836-07

**Authors:** 


PROVINCIAL MEDICAL AND SURGICAL ASSOCIATION.

EASTERN PROVINCIAL MEDICAL AND SURGICAL ASSOCIATION.

In our last Number, (p. 533,) we noticed the establishment of this Association; and we are now happy to announce that it has met with the most marked encouragement and success. The first annual meeting of the Society was held at Ipswich, on Monday, the 6th June, and was attended by upwards of sixty members. Dr. Baird, of Ipswich, officiated as president, and Mr. Crosse, of Norwich, (to whose exertions we believe the Association owes its origin,) as secretary. The following extract from the Report of the Council will show the present state, objects, and purposes of the Society:—" The number of subscriptions paid to the Society's account is 161, and the names of several gentlemen are enrolled from whom no remittance has yet been received, owing to the difficulty of collecting so small an amount,—so that on the present day, when we are not far advanced in the first year of the Society's establishment, the total of its members is not less than 170, and amongst them will be found many of the most venerable of the profession; many of whom have long been distinguished for their literary as well as medical writings, or for their extensive experience and reputation in practice. In the history of every enlightened class of society it has been found, that assembling its members together in large numbers has given a fresh stimulus towards its improvement, and the medical profession is so rich in resources as to profit by such intercourse above most others. Already the fruits of our assembling have ripened and been gathered. Several papers were read at the first meeting of the Council, which are reserved for publication, and others are now ready, some of which will be laid before the present meeting. In order to secure further services, particularly of gentlemen who, from excessive occupation or habits of seclusion, might not otherwise lend their aid, the Council recommend that it should be considered an essential part of the proceedings of each general meeting, to appoint several members to give jointly a report upon some subject referring to the climate, peculiar diseases, statistics, or natural history of the district. It is conceived that no topic is of greater importance to be noticed on this occasion than the terms recommended by the Committee, and the prospect which the Council hopes is held out, 'of effecting a junction with the parent Provincial Association for the publication of Transactions, and holding a general meeting once in a few years;" an arrangement so evidently calculated to produce mutual benefit, combining more of the advantages of union than if the Societies were one, and still retaining to each the chief privileges of a separate existence."

